# Numerical Optimization of Functionally Graded Ti-HAP Material for Tibial Bone Fixation System

**DOI:** 10.3390/ma17215187

**Published:** 2024-10-24

**Authors:** Krzysztof Szymkiewicz

**Affiliations:** Faculty of Mechanical Engineering, Cracow University of Technology, 24 Warszawska, 31-155 Kraków, Poland; krzysztof.szymkiewicz@pk.edu.pl

**Keywords:** functionally graded material, titanium-hydroxyapatite, bone fixation plate, tibia fracture, numerical analysis, micromechanical modeling

## Abstract

Functionally graded materials (FGMs) are heterogeneous composites characterized by outstanding properties. They are built from two or more components with a gradient distribution of chemical composition along a given direction. A promising graded material for biomedical engineering as an implant could be a FGM made of titanium (Ti) and hydroxyapatite (HAP). It would allow us to counteract the difference between the stiffness modulus of pure titanium and bone tissue. Moreover, it can be a good solution to the problem of stress shielding for bone fixation plates made of conventional titanium or steel. The presented paper aims to perform micromechanical modeling and optimization of a functionally Ti-HAP graded plate, followed by numerical analysis of a fractured tibia stabilization system under specific boundary conditions. Finite element analysis was performed using ANSYS Workbench 2021 software. The models of the FGM plate and tibial fixation system were made using the Space Claim tool. The ANSYS software allowed the optimization of the model considered and the selection of the appropriate structural parameters of the FGM Ti-HAP material. In general, the results proved that the osteosynthesis plate built of graded Ti-HAP material resulted in lower bone stress compared to titanium and steel plates. The results obtained confirmed the validity of the design and the possibility to use functionally graded Ti-HAP bone fixation plates.

## 1. Introduction

Titanium and its alloys are well known and widely applied materials with outstanding properties including high specific strength and good corrosion resistance. Therefore, they are especially dedicated for the production of complex parts in various industries like automotive or aviation [1]. They are also used in the manufacture of biomedical components because they effectively absorb external loads from interacting parts and are highly biocompatible. However, one of the important properties in this area is appropriate surface topography, which can contribute to better adhesion of the implant to surrounding bone tissue [2]. Therefore, surface modification processes are usually used to form a biocompatible coating. Among the surface treatment processes that can be used for such applications are plasma spraying, electrochemical modification, laser surface and hybrid processes [2]. Medical parts like endoprosthesis, dental implants and osteosynthesis parts are often coated with hydroxyapatite (HAP) layers, which allow for an improvement in the adhesion of bone tissue to the implant and support bone self-healing processes [3]. Hydroxyapatite is a bioactive and biocompatible material with properties comparable to those of the bone tissue. It plays an important role in the structural strength of human tissue and bone reconstruction as well as bone healing. These advantages enable it to be used in biomedicine for dental or orthopedic implants. However, hydroxyapatite suffer from brittleness and relatively low fracture toughness and tensile strength.

Moreover, the high difference between elastic modulus E values of titanium (~120 GPa), hydroxyapatite and bone (~15 GPa) could contribute to the formation of stress shielding. This is a phenomenon in which the presence of an implant contributes to a reduction in the normal stress on the bone, resulting in lower bone density. As a result, it can lead to implant loosening. Therefore, it seems that the appropriate way to limit material drawbacks and stress shielding might be the production of functionally graded materials (FGMs) constituting the group of unhomogenous composites with outstanding properties. FGMs are composed of at least two components with variable chemical composition, which allow us to obtain a change in properties along a given direction [4,5,6]. They are characterized by better mechanical and functional properties than each component considered separately. This gives the FGMs an advantage over conventional composites and makes them particularly suitable for biomedical applications, where stresses at the implant–tissue interface can be minimized by appropriately optimizing the variation in chemical composition. An additional innovation for this group of materials was the introduction of pores into their structure and the production of porous functionally gradient materials [4,7]. The new class of porous graded materials allow us to reduce their density and as a result to obtain new excellent properties for strictly defined applications.

One of the suitable candidates for biomedical applications could be functionally graded titanium/HAP material. This proposed FGM might better interact with surrounding tissue due to its variable mechanical and physical properties, like density and Young’s modulus. This graded composite is a favorable solution for the mentioned limitations, because the biocompatibility feature of hydroxyapatite is combined with the relatively good mechanical and functional properties of titanium. It is thus possible to obtain a material with the aforementioned properties. A good solution could be the design and optimization of a porous functionally graded material, as it would have a structure and density similar to those of bone, and thus could provide more benefits compared to non-porous FGM materials. Modeling of functionally gradient materials is usually supported by numerical analysis using the finite element method for given boundary conditions and types of analysis. It allows us to select the optimal parameters of the designed material [8].

Jamaludin et al. studied the thermomechanical behavior of a rectangular HAP/Ti graded plate and found that different load conditions of the observed part caused different displacements, temperature profiles and stress distributions [9]. Moreover, they showed that this material could be applied as a high-resistance material due to its good behavior under the influence of the thermal loads.

Sharma et al. considered the effect of thermal and mechanical loads on the deformation and stress of the FGM Ti-HAP plate [10]. They showed that thermal loads have a significant impact on mechanical response of this FGM and that it exhibited high resistance to the thermal stresses. The studies confirmed that it can be applied at higher temperatures. The effect of thermal and mechanical loading on the behavior of the HAP/Ti graded material was also considered using the finite element method [11]. They proved that this graded material can withstand heat and external loads. Furthermore, no delamination of the layers was observed under such loading conditions. Extensive studies of functionally graded plates, with or without porosity, have also been carried out to find generalized relationships describing the properties of the proposed material [12,13,14].

FGMs made from other materials including Al/Al_2_O_3_, Al/ZrO_2_, and Al/SiC have been subjected to structural, mechanical and vibration analysis [15,16,17]. Moreover, Vasiraja et al. studied the non-linear phenomena that occur under external loading of an FGM that consisted of aluminum as the metallic material and alumina and zirconia as the ceramic materials [18]. Static and dynamic analyses were conducted using the finite element method. In this case, many parameters such as component distribution, thickness, and boundary conditions were taken into account. Other research groups have also analyzed functionally graded plates and beams, considering the effect of different functions describing the distribution of components on the mechanical properties (deflection, stress) of the part [19,20].

In biomedical engineering, the issue of stress distribution at the implant–bone tissue interface is still under investigation. Many attempts have been made to perform mechanical analyses of the interface between an implant (for fracture fixation, bone reconstruction, etc.) and the bone tissue. Numerical analyses have been performed on 3D models of the bone and the implant for the real operating conditions of such systems [21,22,23,24,25,26,27,28,29]. Zhang et al. investigated the effect of using a bone fracture plate made of a PEEK-carbon fiber gradient polymer composite on stress shielding at the fracture site. They proved that bone plates made of a gradient composite lead to the formation of relatively lower stresses, including stress shielding, compared to bone plates made of conventional materials, i.e., pure titanium or stainless steel [21]. Similar results were obtained from numerical analyses carried out with bone plates made of titanium alloy, magnesium alloy and PEEK-CFRP polymer composite [22]. The use of a functionally graded Ti-HAP plate, as suggested by [23,24], could be an equally good solution.

The results to date have helped us to establish that design requires a multi-faceted approach, from micro-mechanical modeling, taking into account various factors affecting material properties, to detailed numerical analysis of the behavior of the designed part. Moreover, the knowledge regarding the relationship between the structure of an implant made of a functionally graded composite material and generated stresses and deformations under the influence of real loads is still incomplete. Therefore, the aim of this paper was micromechanical modeling and optimization of a functionally Ti-HAP graded plate and numerical analysis of a fractured tibia stabilization system. Optimization of the Ti-HAP graded plate was carried out by considering two types of structure, including a dense and porous one. Numerical investigations were performed assuming loads and boundary conditions reflecting real conditions. The proposed advanced approach to the modeling and optimization of the Ti-HAP gradient material is novel and has the potential to contribute to the development of biomedical engineering. The results presented in this work pave the way for future research on the design of implants with the required properties and with a high success rate in their application.

## 2. Experimental Procedure

### 2.1. Research Plan

The studies were divided into three parts including modeling of Ti-HAP functionally graded material, optimization of an FGM plate and numerical analysis of a fractured bone stabilization system. Numerical analysis allows for mechanical parameters such as deformation, stress and strain to be measured both quantitatively and qualitatively at a selected location or area. The finite element method is often used in the design of fracture fixation systems with standard plates to evaluate the performance of the model. Based on the results obtained, the durability of the model can then be assessed and the solution implemented in a real application. It was therefore decided to include this method in the submitted paper.

At the beginning, a functionally graded material in the form of a plate was made. The functionally graded titanium-hydroxyapatite (Ti-HAP) material was modeled for different chemical composition coefficients of both elements. Next, the prepared plates were subjected to numerical analysis, aimed at determining the effect of external loads on the stress distribution and deformations. The numerical optimization allowed us to pre-select parameters of the analyzed functionally graded material that would be able to contribute to the formation of the lowest possible stresses and deformations at the implant–surrounding tissue interface. Based on that, a numerical analysis of the tibial–osteosynthesis bone plate system was also carried out.

### 2.2. Material

The material properties used for the numerical analysis of the Ti-HAP graded plate and the model of the fractured tibia bone and osteosynthesis plate are listed in Table 1.

The tibia bone is composed of cortical and trabecular parts. Cortical bone has higher stiffness and strength compared to trabecular bone. Nevertheless, it shows greater brittleness than the latter. Cortical bone has a hard and compact structure, and its function is to carry loads like compression and bending. The trabecular part is made up of spongy tissue, and thus the bone has less structural mass. The cortical bone was defined as the anisotropic material, while the trabecular bone, titanium, hydroxyapatite and stainless steel were the isotropic materials. Pure titanium and stainless steel were selected for comparison purposes as typical materials used in the manufacture of osteosynthesis plates. The screws were made of pure titanium.

### 2.3. Modeling of Titanium/Hydroxyapatite FGM Plate

**Figure 1:**
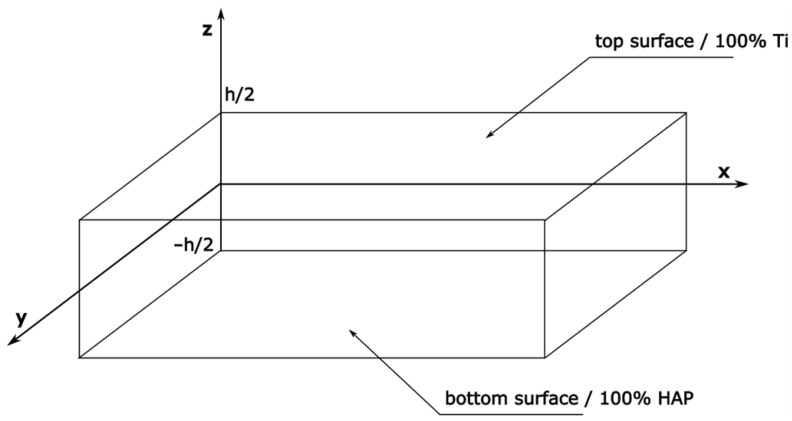
Model of a Ti/HAP functionally graded plate in the coordinate system.

**Figure 2:**
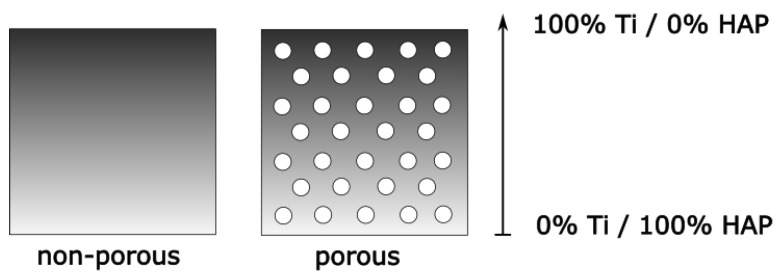
Cross-sectional scheme of the considered non-porous and porous FGM plates.

The rectangular-shape plate made of the Ti/HAP functionally graded material was characterized by a layered structure and it was formed of 10 and 20 layers. Our approach was adopted to assess the effect of the number of layers on the mechanical parameters analyzed, including deformation and stresses. The FGM plate was modeled with different chemical compositions of components along the cross-section of the sample. A scheme of the modeled plate is presented in Figure 1. The length, width and thickness of the Ti-HAP plate were 100, 10 [mm] and 4 [mm], respectively. The thickness of the plate is designated as “h”. It was assumed that top surface layer (z = h/2) of FGM plate was rich in the metallic compound titanium (100% Ti), while its the bottom surface layer (z = −h/2) was made only of ceramic hydroxyapatite (100% HAP). The “z” value describes the distance between the middle part of plate and its top and bottom surfaces. This part of the paper is concentrated on the analysis of plates with non-porous and porous structures. A porous plate was characterized by an even distribution of pores. The scheme of the considered plate model is shown in Figure 2.

The effective material properties of individual layers of the FGM plate, including Young’s modulus, density and Poisson’s coefficient, were calculated with the help of the rule of mixture. These parameters were determined using the following formula:

- for the plate without porosity,
  P = (Pt − Pb) Vf + Pb(1)
- for the plate with porosity,
  (2) $$P\;=\;(Pt\;-\;Pb)\;Vf\;+\;Pb\;-\frac{\alpha }{2}\left(Pt+Pb\right)$$
  where Pt and Pb are the material properties of the top and bottom surface layer, Vf is the volume fraction of the plate and α is the porosity parameter in the range of 0 up to 1. α = 0 corresponds to non-porous FGM plate.

The above relationship shows that the material properties of a given layer in the FG plate depend on the volume fraction, which can be determined with the use of the power law equation below:

Vf = (z/h + 0.5)^n^ (3)

where the n-parameter defining the material distribution in individual layers depends on the thickness, which is described as the volume fraction exponent.

Based on the above mentioned general equations describing material properties, the calculations for the non-porous and porous plate were performed as below:

- for the plate without porosity,
  (4) $$E\left(z\right)=\left(E_{Ti}-E_{HAP}\right){\left(\frac{1}{2}+\frac{z}{h}\right)}^{n}+E_{HAP}$$
  (5) $$\nu \left(z\right)=\left(\nu_{Ti}-\nu_{HAP}\right){\left(\frac{1}{2}+\frac{z}{h}\right)}^{n}+\nu_{HAP}$$
  (6) $$\rho \left(z\right)=\left(\rho_{Ti}-\rho_{HAP}\right){\left(\frac{1}{2}+\frac{z}{h}\right)}^{n}+\rho_{HAP}$$
- for the plate with porosity,
  (7) $$E\left(z\right)=\left(E_{Ti}-E_{HAP}\right){\left(\frac{1}{2}+\frac{z}{h}\right)}^{n}+E_{HAP}-\frac{\alpha }{2}\left(E_{Ti}+E_{HAP}\right)$$
  (8) $$\nu \left(z\right)=\left(\nu_{Ti}-\nu_{HAP}\right){\left(\frac{1}{2}+\frac{z}{h}\right)}^{n}+\nu_{HAP}-\frac{\alpha }{2}\left(E_{Ti}+E_{HAP}\right)$$
  (9) $$\rho \left(z\right)=\left(\rho_{Ti}-\rho_{HAP}\right){\left(\frac{1}{2}+\frac{z}{h}\right)}^{n}+\rho_{HAP}-\frac{\alpha }{2}\left(E_{Ti}+E_{HAP}\right)$$
  where α is equal to 0.1, 0.2 and 0.3.

**Figure 3:**
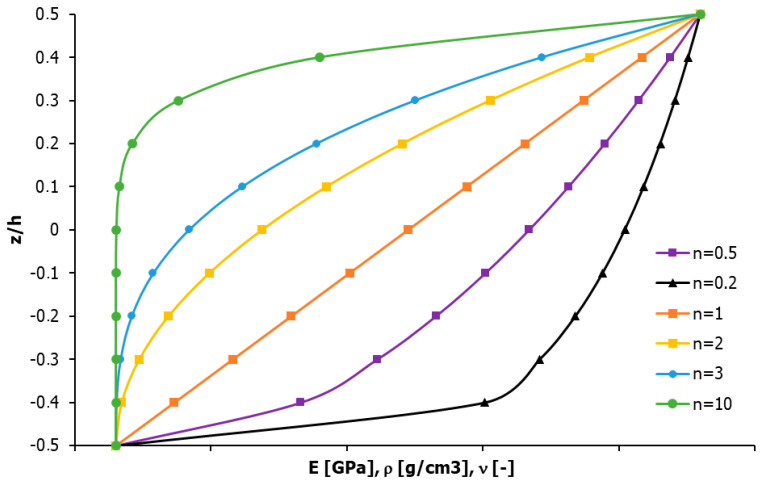
Graph of the property changes along the cross-section of the plate depending on the n-coefficient of the power law.

Calculations of the effective properties of the FGM material were performed for six values of the n-parameter of the power law, including n = {0.5, 0.2, 1, 2, 3, 10}. The distribution of the changes in the properties of the plate under consideration in the cross-section is shown in Figure 3.

### 2.4. Numerical Analysis of Ti-HAP FGM Plate

**Figure 4:**
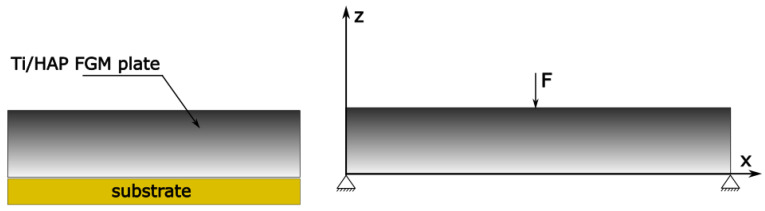
Considered Ti/HAP graded model with boundary conditions and loads.

Numerical analysis was carried out with the use of Ansys Workbench 2021 software. The model was subjected to a force applied in a perpendicular direction to the plate surface, as shown in Figure 4. The plate was uniformly loaded with a load of 800 [N], corresponding to a typical adult body weight (80 kg). Both ends of the plate were fixed. The analysis was performed for different mesh density values, aimed at a convergent validation of the obtained results.

The prepared models of the functionally graded non-porous and porous plate with different power law n-parameters including 0.2, 0.5, 1, 2, 3, and 10 were subjected to a meshing process. The numerical model was divided into a finite number of individual parts with given sizes, including 0.2, 0.3, 0.4, 0.5, 1, 2. All used parameters of the studied model and boundary conditions are summarized in Table 2.

After numerical analysis, the optimal model parameters were selected and implemented for numerical studies of the bone fixation plate.

### 2.5. Modeling and Numerical Analysis of a Fixation Plate for a Fractured Tibia

**Figure 5:**
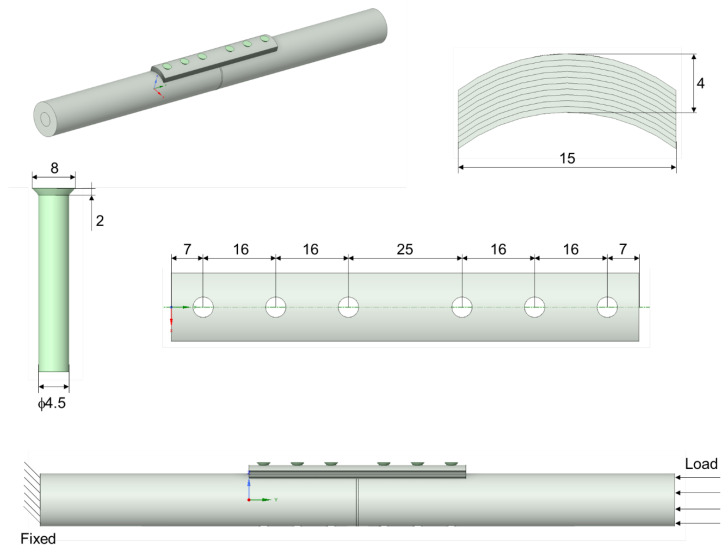
Scheme of plate, bone and screw geometry.

In the second part of the study, models of the tibia bone, the osteosynthesis plate and the screws connecting the two parts were made. The models were designed with the help of the Space Claim tool of the Ansys Workbench 2021 software. The bone-to-plate connection scheme is shown in Figure 5.

The tibia model was simplified to the shape of a cylinder with a length of 300 mm, composed of two parts, including an inner cylinder with a diameter of 10 mm corresponding to the trabecular bone, and an outer cylinder with an external diameter of 25 mm, representing the cortical bone. The fracture space dividing the bone into two parts was the same thickness of 1 mm in all cases analyzed. The fracture space contained callus tissue, which has different mechanical properties depending on the degree of bone healing. Three phases of bone healing, including 1%, 50% and 75%, were considered [21]. Callus properties are included in Table 3.

The osteosynthesis plate with functionally gradient properties was characterized by a layered structure. Two types of FGM plate with a non-porous and porous structure were analyzed. Their dimensions were a 103 mm length and a 4 mm thickness, and the curved cross-section had a radius of 13.7 mm. The plates had six screw holes. The titanium screws were 4.5 mm in diameter. Two cases of contact between the plate and bone were analyzed in this study, including contact (no-gap) and non-contact (1 mm gap) connection.

Tests were performed for a bone plate with optimized properties based on a numerical analysis of a simplified plate (test description in Section 2.3). For comparison purposes, numerical analyses were also carried out for a bone plate made of pure titanium and stainless steel.

The bone model was fixed on one side and a load was applied on the other side. The load on the model was dependent on the degree of healing of the bone, i.e., 8 kg for 1% healing, 80 kg for 50% healing and 160 kg for 75% healing. The load applied to the bone corresponds to the time that elapses after osteosynthesis surgery. Immediately after surgery, the main load is taken up by the plate, and then, over time, the healed bone can take up more load. This approach has been described and analyzed in [21].

## 3. Results

### 3.1. Overview of the Numerical Optimization of the Ti-HAP FGM Plate

The deflection parameter is an important parameter in terms of the potential use of the optimized material as a plate for bone stabilization. The function of the plate is to transfer loads and to stiffen the bone. Therefore, the lowest possible plate deformation is expected, which may affect the bone–plate junction. Table 4 shows the deflection of the non-porous and porous plate depending on the considered N coefficients of variation in the chemical composition. The response of the FGM Ti-HAP plate, which was composed of ten layers, was deformation under loading. The average value of plate deflection was ~0.4 mm. An increase in the number of layers in the plate to twenty resulted in a slight increase in deformation (about 1%). The lowest deformation was obtained for the non-porous graded material. The pore’s introduction to the structure of the functionally graded plate caused an increase in material deflection, which might have been caused by the lower material density. The largest deformation of the plate was noted for the model with the highest degree of porosity (α = 0.3). It was observed that the increase in the analyzed parameter was non-linear. A change in the coefficient of variation in material properties (from 0.2 to 10) contributed to an average increase in the deformation of the FGM plate of 2–9%. The lowest values of plate deflection were obtained for the non-porous material with an N coefficient of 0.2. However, it should be noted that porous structures can favorably influence the processes of bone ingrowth into the implant and reduce stress shielding due to the material’s relatively lower structural mass and its lower stiffness close to the bone. Estimating the optimal porosity coefficient of the material could allow better tissue growth around the implant while maintaining adequate mechanical properties.

**Figure 6:**
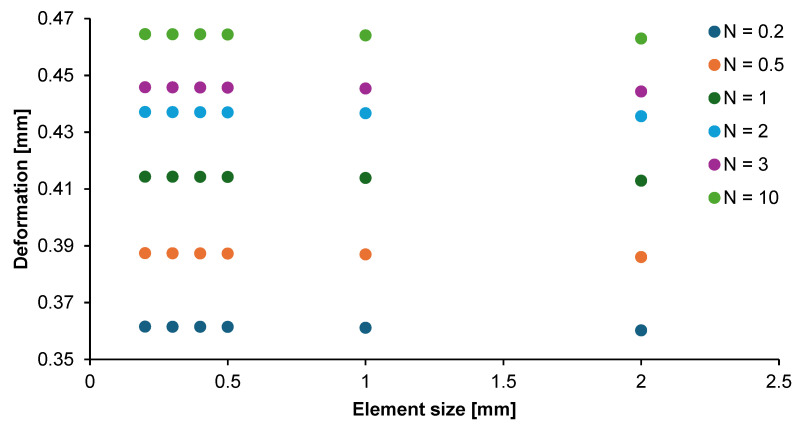
Deformation variability of graded non-porous plate for a given element size.

The convergence study showed how the mesh density of the model affected the accuracy of the results obtained. The parameterization allowed the determination of the average solution error and the optimum element size. Figure 6 contains a graph of the dependence of the FGM plate deformation on the mesh size of the element. The analysis showed that the model with an element size of up to 0.5 mm gave the most convergent results, and beyond this, that size leads to significant differences in results. 

In the next parts of paper, the results are presented for the model built from elements with a size of 0.5.

**Figure 7:**
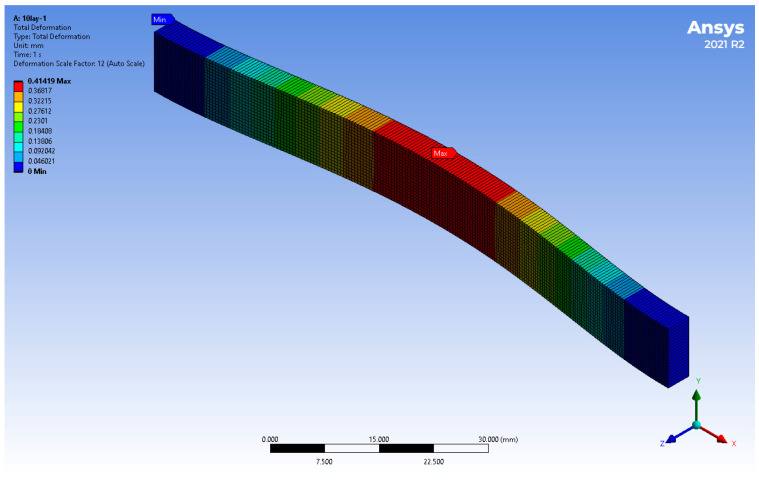
Total deformation of the Ti/HAP graded plate (ex. 10-layered non-porous plate).

The deformation distribution of the 10-layered FGM non-porous plate is shown in Figure 7. The largest deformation was observed in the middle part of the plate. The deformation from this area decreased along the length of the plate toward its restraints, which is consistent with the adopted boundary conditions.

**Figure 8:**
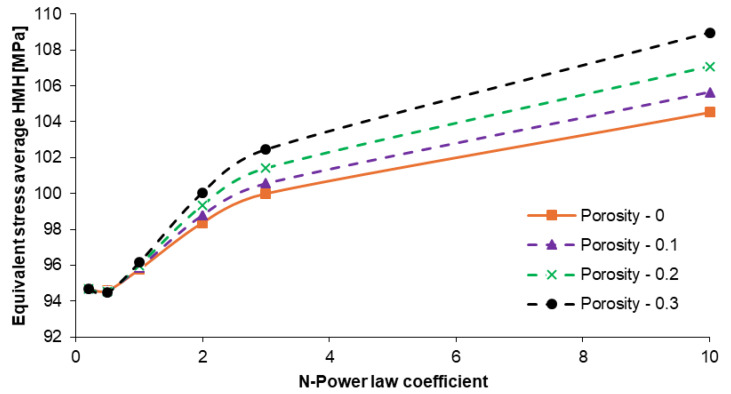
Graph of equivalent stress average HMH changes in dependence on the N-power law coefficient for the 10-layered FGM Ti-HAP non-porous and porous plates.

The average equivalent stresses for both types of plates with different N–power law coefficients are presented in Figure 8. As a result of the part load, the formed stress was equal to 110 [MPa]. The lowest stress (~96 MPa) was characterized by an N coefficient of up to 1. Stress differences for these models were in the range of 2 MPa. Increasing the coefficient resulted in an increase in plate stress. Moreover, the changes in the stress had a non-linear course. The plate model built with an N coefficient of 3 and above resulted in linear stress increases ranging from ~100 up to 109 MPa. The non-porous material exhibited less susceptibility to stress under external loads. The highest average stresses were observed for all the plates analyzed with a porosity coefficient of α = 0.3.

Table 5 contains the average values of stress forming in the non-porous and porous Ti-HAP graded plates. The performed numerical analysis indicated that increasing the number of layers in the plate up to twenty does not significantly affect the change in the reduced HMH stress value. The highest values of stress was in the plate with an N coefficient of 10. The obtained values of the considered parameter were approximately 106 MPa. Moreover, the analysis of the obtained results showed that porosity does not cause a change in formed stresses for materials with an N-power law coefficient of up to 2. Significant differences in stress were observed for the porous plate with a higher N coefficient. It was observed that strains of the plate rise with an increasing n-parameter, as can be seen from Table 6. The average values of plate strains were in the range of 8.8 × 10^4^ to 17 × 10^4^ [mm/mm].

**Figure 9:**
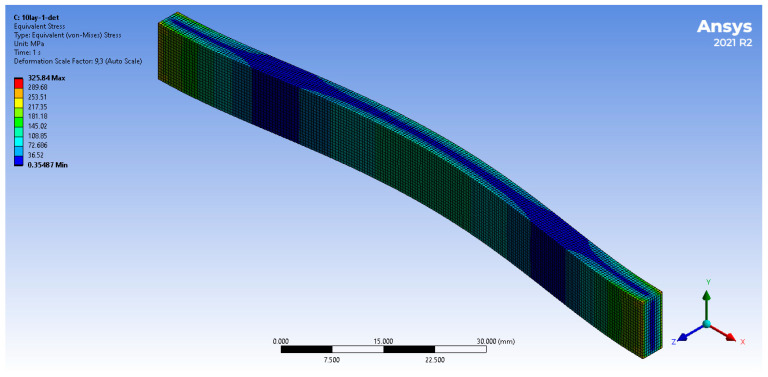
Equivalent stress distribution of the Ti/HAP graded 10-layer dense plate.

Figure 9 shows the general stress distribution of a non-porous FGM plate with an N of 1. As is shown, lowest stress values were noted at the distance of approximately 0.25 in length of the FGM plate from the restraint and also in its central part along the length of the plate. The average values of the stresses occurred at the point of load application and at the bottom of the plate, while the highest values of the analyzed parameter were observed at both ends of the plate.

The forms of the resulting stresses and strains in the FGM model were similar to those obtained in the research of Jamaludin et al. and Kar et al. [9,11]. The obtained results of numerical analysis helped to establish that models built with 10 layers with an N-power law coefficient up to 1 exhibit relatively low stresses and deformation for the considered loading system. Such models with optimized structures should be welcomed in the case of their application as medical implants. However, additional mechanical analysis the selected structural parameters of the material is needed. The detailed results of FEA analysis of optimized non-porous and porous plates for different boundary conditions and loads are described in Section 3.2.

### 3.2. Numerical Analysis of the Optimized Ti-HAP FGM Plate

**Figure 10:**
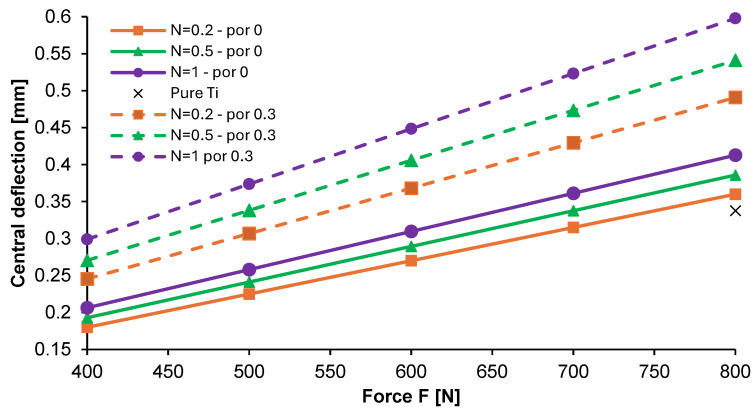
Deflection of the Ti-HAP graded material along the x-direction of the plate.

Figure 10 shows the effect of external force on the central deflection of the non-porous and porous plate with an N-coefficient up to 1. An increase in loading force led to a rise in the total deformation of both of the considered plates. The porous plate was more deformed. Moreover, the change in the N-coefficient caused a slight increase in deflection of 7% on average. The increase in plate deflection with load was linear for all cases considered. The results obtained for the dense graded plate were consistent with those in [10].

**Figure 11:**
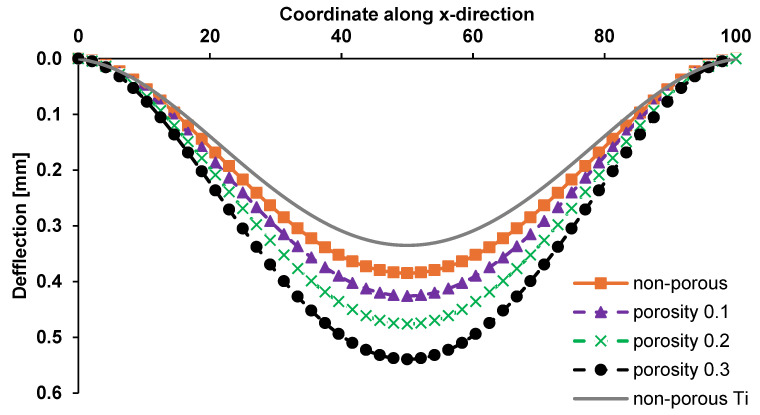
Effect of porosity on central deflection of Ti-HAP functionally graded plate.

The deflection distribution along the length of the Ti-HAP graded plate is presented in Figure 11. The change in plate deformation had a sinusoidal waveform in all cases. The maximum values of deformation were observed in the middle part of plate. The bending distribution of the graded plate was compared with that of the non-porous plate composed of pure titanium. The latter was characterized by the lowest deflection, resulting due to the greater stiffness of the material.

**Figure 12:**
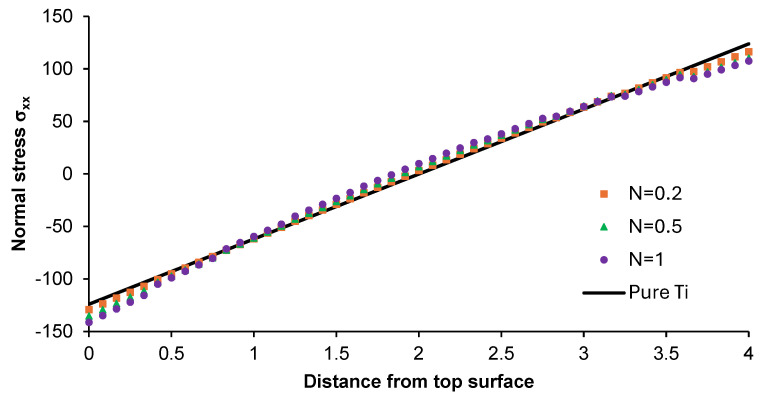
Normal stress σ_xx_ distribution along the z-direction from the top surface of the Ti-HAP graded plate under the influence of load.

**Figure 13:**
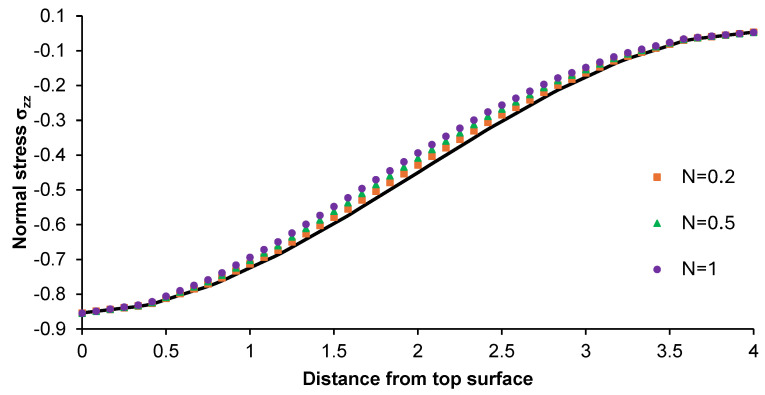
Normal stress σ_zz_ distribution along the z-direction from the top surface of the Ti-HAP graded plate under the influence of load.

Figure 12 and Figure 13 present the distribution of normal stress σ_xx_ and σ_zz_ along the z-direction from the top surface of the non-porous graded plate for different N-coefficients. The parameters analyzed were compared with the normal stress distribution for the pure titanium plate.

The change in σ_xx_ was close to sinusoidal course, while slightly changes in the considered parameter were observed for the graded plate with an N-power law coefficient of 0.2. The results were similar to those on stresses formed in the pure titanium plate. For the latter, a linear increase in stress along the plate thickness was observed. The highest differences in stress values were observed for the material with an N-power law coefficient of 1. In the case of normal stress σ_zz_ for each considered plate, a non-linear growth in stress was observed. Moreover, the values of this parameter were negative along the thickness of the part. The normal stress values were close to each other in the top and bottom parts of the functionally graded plate, while higher differences in this parameter were registered in its central part.

**Figure 14:**
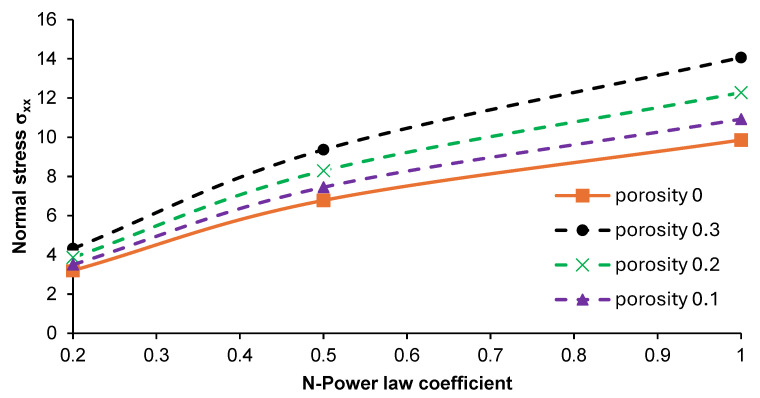
Effect of the N-power law coefficient on the normal stress σ_xx_ in the middle part of the porous and non-porous plates at the point (x/2, y/2, z).

**Figure 15:**
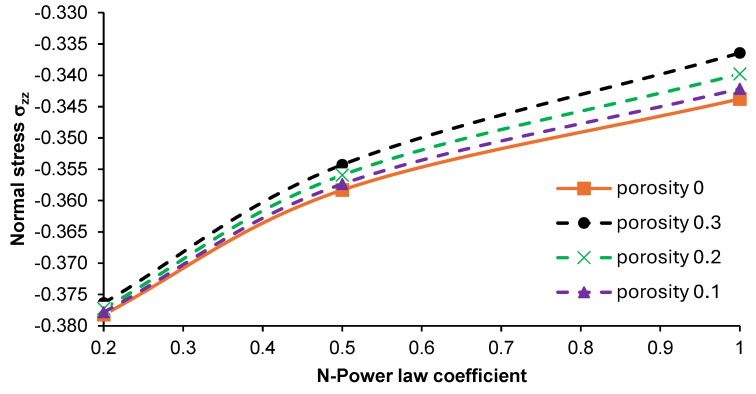
Effect of the N-power law coefficient on the normal stress σ_zz_ in the middle part of the non-porous and porous plates at the point (x/2, y/2, z).

The obtained results of the investigations helped us to establish that the central part of the plate should be subjected to further analysis due to the differences in stresses produced. The effect of N-coefficient changes on the distribution of normal stress σ_xx_ and σ_zz_ in the given area of the part was considered, as shown in Figure 14. The performed analysis revealed that introducing porosity into the material resulted in an increase in normal stress σ_xx_ in the graded material. The highest stress values were noted for the plate with an N-power law coefficient of 1. An increase in the N-parameter from 0.2 up to 1 led to an approximately two-fold increase in normal stress σ_xx_ in the case of each analyzed plate. The normal stress in the z-direction (σ_zz_) showed negative values, and the increases in this parameter were relatively small, as illustrated in Figure 15.

**Figure 16:**
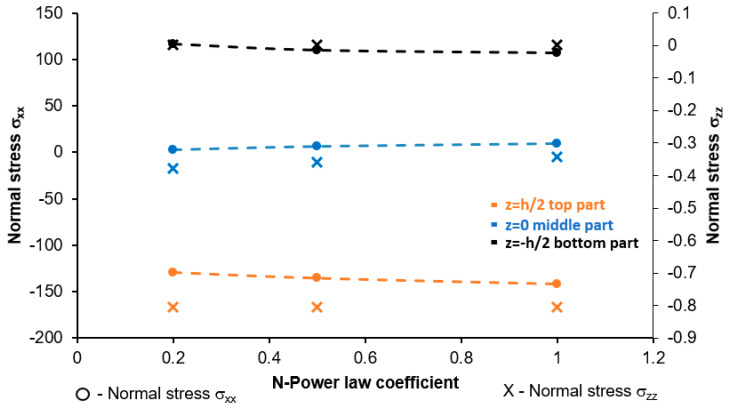
Normal stresses σ_xx_ and σ_zz_ of the Ti-HAP graded non-porous plate in the central part.

The distribution of normal stresses σ_xx_ and σ_zz_ in the top, middle and bottom (z = −h/2, 0, h/2) parts of cross-section of the plate depending on the N-coefficient is presented in Figure 16. An increase in N-coefficient did not result in any significant changes in both of the analyzed normal stresses. The highest normal stress was observed in the bottom part of the plate rich in hydroxyapatite, while the lowest was noted in the upper part of the plate composed of pure titanium.

**Figure 17:**
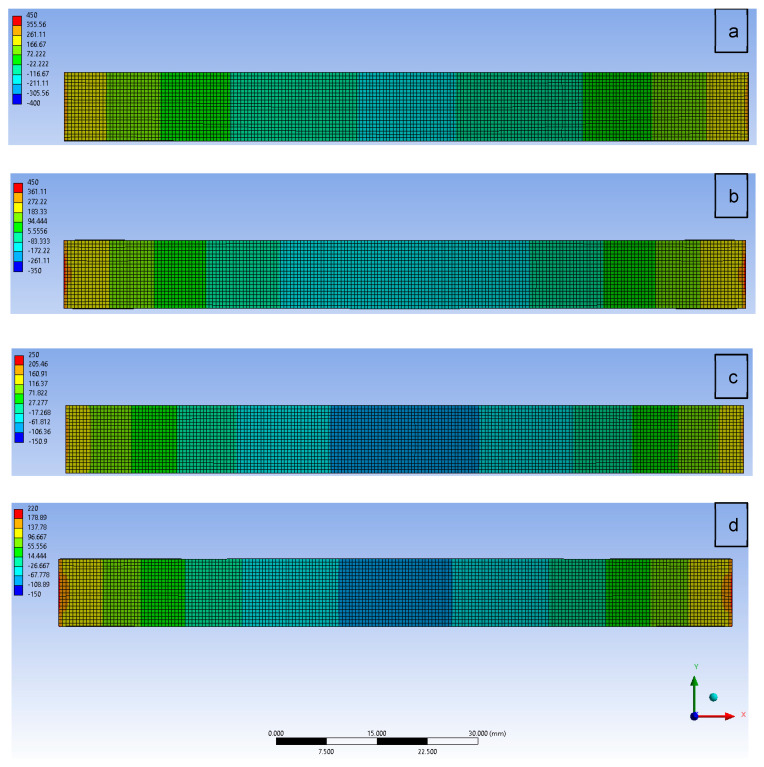
Normal stress σ_xx_ distribution on the top surface of Ti-HAP graded plates with porosity coefficients of (**a**) α = 0, (**b**) α = 0.1, (**c**) α = 0.2, and (**d**) α = 0.3.

The effect of plate porosity on normal stress σ_xx_ distribution on the top surface (100% of Ti) of the plate is shown in Figure 17. As can be observed, the stress distribution changed along the length of the functionally graded plate. The highest normal stress was produced at the ends of the plate, while the lowest one was noted in its central area. The largest differences in the considered normal stress appeared in the non-porous material. An increase in the porosity coefficient up to 0.3 resulted in a slightly lowered stress and its more even distribution on the top surface of the FGM plate.

The optimization results of the simplified functionally graded plate showed that the deformation and stresses of the component were directly influenced by the material structure and the coefficient of variation in the chemical composition. A comparative analysis of the discussed results with the literature data on the static analysis of such materials confirmed their correctness [9,12,16].

Taking into account the proposed use of such plates as medical implants, it is necessary to achieve the lowest possible stresses and strains at the implant–bone tissue interface that could affect the durability of the joint. In addition, the analyses carried out showed that functionally graded non-porous and porous Ti-HAP plates made of 10 layers with a ratio of N up to 1 exhibit appropriate properties for the designed medical application. Therefore, a numerical optimization of the plate tibia bone fixation system was performed for materials with the selected parameters, and the results are presented in Section 3.3.

### 3.3. Numerical Analysis of Osteosynthesis Plate–Fractured Tibia Bone System

#### Results Overview

Table 7 presents the calculated maximum stress and total deformation of the model with the Ti-HAP FGM, titanium and stainless steel of plate. Deformation of the tibia bone osteosynthesis FGM plate was similar to the deformation of the model with pure titanium and stainless steel plates. Deformation values varied up to 6% for all callus tissue conditions considered, corresponding to the percentage of bone healing.

The lowest stress values of the model analyzed were observed for the FGM plate model with a coefficient of variation N of 1. More favourable results were obtained for a plate with a porous structure. The use of a plate made of stainless steel and titanium contributed to the formation of higher stresses depending on the percentage of bone healing. Therefore, the best case for further analysis seems to be the use of an FGM porous plate with a parameter N of 1.

**Figure 18:**
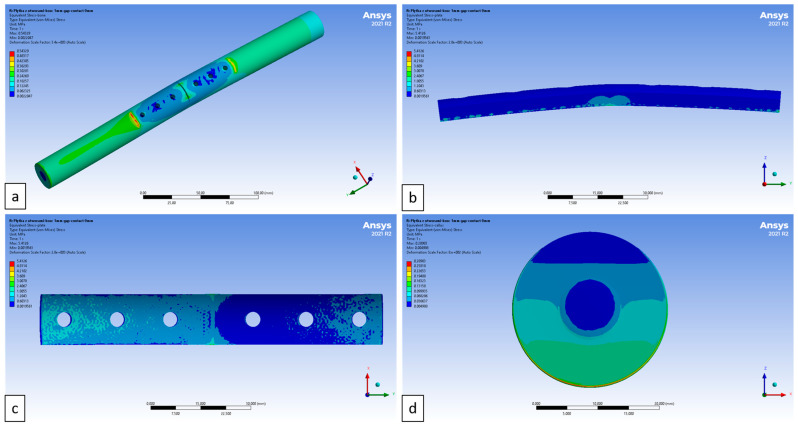
Maps of stress distribution in (**a**) bone, (**b**,**c**) plate and (**d**) callus.

Stress distribution maps of the bone, plate and tissue models are shown in Figure 18. It can be observed that the maximum stress values in the bone model occurred in the bone–plate interface area along the shorter side of the plate, while the lowest values were noted at the screw holes, which is also consistent with the considered loading model. A relatively low stress distribution was observed for the fracture plate at the bone interface. A uniform stress distribution was found on the callus tissue, increasing from the plate–bone interface to the lower part of the plate.

**Figure 19:**
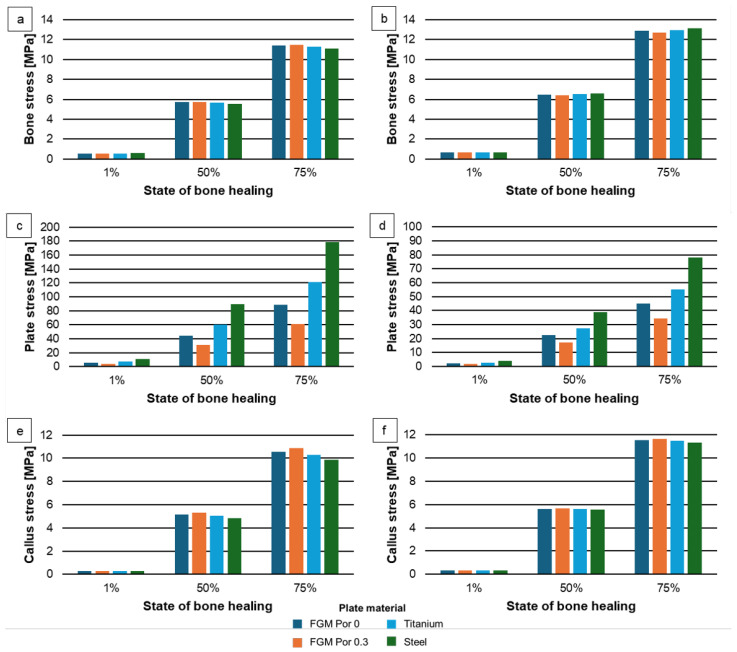
Maximum stress of bone, plate and callus for different states of bone healing for contact (**a**,**c**,**e**) and non-contact (**b**,**d**,**f**) plate-to-bone connection.

A graph of maximum stress of the bone, plate and callus for different states of bone healing is presented in Figure 19. The bone stress values obtained for the non-porous and porous FGM plates for the direct-connection bone-to-plate system were similar. The maximum stress value was obtained for the model with 75% of bone healing, but then the tissue took on a higher load. The maximum stress of the bone was approximately 11.5 MPa for the model with the porous FGM plate. The lowest stress values were observed for the model corresponding to the initial state after the treatment.

The formed stresses in the model as a result of its loading were the highest for the plate made of conventional materials compared to Ti-HAP graded material. In the case of the direct bone plate–junction, the average stress of the Ti plate reached values of approximately 8, 60 and 120 MPa for a bone model with a healing state of 1%, 50% and 75%, respectively. Twice, stresses were observed for the non-contact bone-to-plate case (Figure 19c,d).

The obtained maximum stress of the FGM plate (~90 MPa) for the considered load system did not exceed the strength of HAP, which supports the possibility of their use in practice. Moreover, the 0porous plate for osteosynthesis contributed to the formation of lower stresses compared to parts with a dense structure. Therefore, this plate structure could be favorable for the proposed application.

The callus stresses were relatively close to those obtained for the bone tissue. The highest stress values were observed for the model with 75% of bone healing (~10 MPa), while the lowest values were obtained for the initial state after bone fixation surgery (1% of bone healing).

The use of non-contact between the osteosynthesis plate and bone (1 mm of gap) caused an increase in stress in the bone and callus in the range of 7% up to 25% for all analyzed cases. Comparing models with a plate made of FGM and conventional material, greater increases in stress were observed for the latter. Moreover, the lack of connection between the plate and the bone affected the stresses at the border of the two parts, which were lower compared to those in the case with direct connection them (no-gap plate–bone). These decreases ranged from 35% to 55% in the model with the FGM plate.

## 4. Discussion

Osteosynthesis plates are commonly used for the treatment of bone tissue fractures, providing appropriate stabilization of the fracture. They also reduce the load on the bone, contributing to the correct healing process of the tissue. Bone plates should have good strength properties, corrosion resistance and high biocompatibility. Adequate bone fixation with a plate leads to correct tissue growth in the fractured area and the patient’s recovery. Otherwise, improperly healed bone may contribute to pain. Among the metallic plates are those made of titanium alloy (Young’s modulus E = 110 GPa), which have replaced the stainless steels (E = 210 GPa) previously used. Despite their better mechanical properties, i.e., high strength or lower Young’s modulus, as well as the good biocompatibility of titanium alloys compared to that of steel, they suffer from relatively poor wear resistance and fatigue strength. Moreover, these materials have a high modulus of stiffness compared to that of bone. These disadvantages can lead to undesirable stress shielding effects, leading to implant loosening. Moreover, clinical studies focusing on the use of titanium alloys in medicine have shown a relatively high success rate (>90%). However, the disadvantages, such as the poor abrasive properties, high stiffness and negative impact on human health, of alloy additives such as aluminum and vanadium are still being highlighted. It is therefore important to carry out further studies to find an optimal solution that would improve the usability of titanium-based materials. Functionally graded materials with excellent properties, which have been the subject of much research, appear to be a promising solution to overcome these drawbacks [15,16,20]. These are materials composed of at least two materials with a gradual change in chemical composition across the cross-section, resulting in variable properties, i.e., stiffness and density. This approach can lead to a better interaction at the implant–bone tissue interface. These materials have a major advantage over conventional monolithic materials. An example of such a material for medicine is functionally gradient titanium-hydroxyapatite, which has a combination of the good mechanical properties of titanium and biocompatible hydroxyapatite, showing similar properties to bone tissue [9,10]. Materials based on these components have been extensively investigated experimentally to assess their potential use in clinical practice as dental or orthopedic implants [30,31]. Analyses have confirmed that Ti-HAP materials have good osteointegration and biocompatibility properties. They are also characterized by the ability to form new bone tissue and could therefore be successfully used in practice.

The subject of this paper was to design and optimize functionally graded plates used as bone fixation plates. An optimization analysis was performed for a simplified Ti-HAP gradient plate and an advanced plate fixation system of a fractured tibia. For the first case, a plate with different coefficients of variation in chemical composition and a dense and porous structure was considered. For the second case, the effect of different bone healing states, loading and type of implant–tissue interface on the behavior of the model was analyzed. This approach allowed both models to be verified in detail and the optimum solution to be estimated.

The studies indicated that osteosynthesis plates made of Ti-HAP FGM with a stiffness similar to that of bone tissue near the bone–implant interface contribute to even stress distribution in the bone. They can effectively reduce the formation of stress shielding during bone healing, which could affect the quality of the implant–tissue connection. In addition, the formation of stresses in the model (fractured bone and plate) depends on the distribution of the chemical composition of the FGM plate and its structure (non-porous and porous). It was also found that non-contact connection at the plate–bone interface directly affects the reduction in stress and deformation values, and thus is more favorable for the designed applications.

It should be noted that such a broad approach to modeling and optimizing metal-ceramic FGM plates that stiffen fractured bone by analyzing the effect of porosity on plate response has not been taken this far yet.

So far, numerical simulations of the bone fixation plate (Ti-HAP FGM) have been realized; it has been found that this type of material provides improved stabilization of the plate–fractured bone connection system and the stress shielding phenomenon is reduced in all stages of bone healing [23,27]. These conclusions are consistent with the results obtained in the presented work. Similar studies of Ti-HAP composites also highlighted that the positive effects of the good mechanical properties of titanium and of the osseointegration properties of hydroxyapatite contribute to better bone regeneration [24,32].

In addition to metal–ceramic plates, those made of polymer composites (i.e., PEEK-CFRP, CF/epoxy, GF/epoxy,) or magnesium alloys have also been studied using numerical mechanical analysis of the model, taking into account the bone healing process [21,22,25,26,28]. Low-density composite plates have also been shown to play their intended role by achieving a more uniform stress distribution in the bone fracture area. Thus, they could be better materials than metallic plates.

Nevertheless, it should be noted that FGMs based on the titanium and hydroxyapatite seem to be a suitable choice for medical implant applications, as titanium is responsible for strength, while hydroxyapatite provides adequate bioactivity during the bone healing process. Therefore, these materials should be subjected to further research.

## 5. Conclusions

An attempt was made to optimize a functionally graded Ti-HAP plate for the stabilization of a fractured tibia under different boundary conditions. This study was carried out using the finite element method. To sum up, the presented results of investigations allowed us to formulate the following conclusions:

1. The number of layers in the structure of the FG material does not have a significant impact on its mechanical properties.
2. A Ti-HAP functionally graded model made of 10 layers and with an N-power law of up to 1 ensures the formation of the lowest stresses and deformations.
3. The introduction of porosity into the structure of the FGM material reduces its mechanical properties, but could be welcomed in biomedical applications in the case of a growth of tissue surrounding the implant.
4. The best solution is to use a bone fracture plate made of functionally graded Ti-HAP material with an N-power law coefficient of 1 and a porous or dense structure (in favor of the former).
5. The lack of a direct plate–bone fracture connection contributes to the formation of lower stresses and strains, which are more beneficial for the intended application.

The chosen test method is characterized by limitations due to the need for simplifications in the modeling and analysis stages. Nevertheless, by performing a series of numerical tests for models of different complexities and loading systems, optimal results can be obtained, as shown by the results presented in this paper. The obtained findings can be helpful to the further considerations and analysis in this area. However, the research should concentrate on numerical simulations of the effect of pore distribution in the model and of the size of pores on mechanical response. Studies could be extended to include numerical analyses of plate models made of other materials under consideration, such as biocompatible polymer composite or magnesium alloys. This approach would provide a complete overview of the potential use of materials as bone fixation plates. The result of this study should be validated through experimental tests. Based on multi-aspect research, it would be possible to attempt to create a constitutive model of bone fixation plates made from FGM material, which is plannedfor future research.

## Figures and Tables

**Table 1:** Material properties of plate, screws, cortical and trabecular bones.

No.	Material	Density δ [g/cm^3^]	Young’s Modulus E [GPa]	Poisson’s Ratio ν
1	Ti	4.5	116	0.34
2	HAP	3.15	73	0.28
3	Stainless steel	7.9	193	0.31
4	Cortical bone	1.8	E_x_—7 E_y_—18.4 E_z_—8.5	v_xy_—0.099 v_yz_—0.065 v_xz_—0.141
5	Trabecular bone	0.37	1.02	0.225

**Table 2:** Parameters of considered FGM model and boundary conditions.

Number of FGM Layers	Porosity Coefficient α	Power Law n-Coefficient	Load [N]	Mesh Size [mm]
10	0 0.1 0.2 0.3	0.2 0.5 1 2 3 10	800	0.2 0.3 0.4 0.5 1 2
20				

**Table 3:** Properties of callus in different states of bone healing.

Material	Density δ [g/cm^3^]	Young’s Modulus E [GPa]	Poisson’s Ratio
Callus (1% healing)	0.731	0.2	0.3
Callus (50% healing)	1.434	10	0.3
Callus (75% healing)	1.699	15	0.3

**Table 4:** Deformation of FGM plate (10 layers) for different porosity coefficients.

N-Power Law	Deformation [mm]				
	FGM—10 Layers				FGM—20 Layers
	α = 0.1	α = 0.2	α = 0.3	α = 0	α = 0
0.2	0.39662	0.43916	0.49161	0.3614	-
0.5	0.42812	0.47839	0.5418	0.38725	0.39194
1	0.46165	0.52128	0.59867	0.41419	0.41902
2	0.49042	0.55886	0.64998	0.43695	-
3	0.50049	0.57308	0.66934	0.44566	0.45064
10	0.52439	0.60214	0.70726	0.46437	-
Average	0.46695	0.52882	0.60978	0.41830	0.42053

**Table 5:** Equivalent stress average HMH of porous and non-porous FGM plates.

N-Power Law	Equivalent Stress Average HMH [MPa]				
	10 Layers				20 Layers
	α = 0.1	α = 0.2	α = 0.3	α = 0	α = 0
0.2	94.703	94.695	94.674	94.701	-
0.5	94.573	94.539	94.483	94.59	93.72
1	95.869	95.997	96.151	95.76	95.669
2	98.796	99.337	100.05	98.37	-
3	100.56	101.41	102.46	100	101.18
10	105.65	107.08	108.97	104.53	-

**Table 6:** Equivalent strain average of porous and non-porous FGM plates.

N-Power Law	Equivalent Strain Average [mm/mm] × 10^4^				
	10 Layers				20 Layers
	α = 0.1	α = 0.2	α = 0.3	α = 0	α = 0
0.2	9.63	10.65	11.92	8.78	-
0.5	10.40	11.61	13.15	9.41	9.53
1	11.21	12.65	14.53	10.06	10.18
2	11.89	13.54	15.75	10.60	-
3	12.13	13.87	16.19	10.80	10.92
10	12.68	14.54	17.07	11.24	-

**Table 7:** Maximum stress and total deformation of the model (direct connection of bone/plate) for different plate materials and bone healing degrees.

	Plate FGM N = 0.2		Plate FGM N = 0.5		Plate FGM N = 1		Titanium Plate	Stainless Steel Plate
	α = 0	α = 0.3	α = 0	α = 0.3	α = 0	α = 0.3		
Max stress of model 1% healing [MPa]	7	5	6	4	5	4	7	11
Max stress of model 50% healing [MPa]	55	41	49	36	44	31	61	90
Max stress of model 75% healing [MPa]	109	83	98	71	89	61	121	179
Total deformation1% healing [mm]	0.006	0.006	0.006	0.006	0.006	0.006	0.006	0.005
Total deformation 50% healing [mm]	0.044	0.045	0.045	0.046	0.045	0.046	0.044	0.042
Total deformation 75% healing [mm]	0.088	0.089	0.089	0.091	0.090	0.092	0.087	0.085

## Data Availability

The original contributions presented in the study are included in the article, further inquiries can be directed to the corresponding author.
